# Food from the Wild—Roles and Values of Wild Edible Plants and Fungi

**DOI:** 10.3390/foods13060818

**Published:** 2024-03-07

**Authors:** Luis Catarino, Maria Manuel Romeiras, Ângela Fernandes

**Affiliations:** 1 Centre for Ecology, Evolution and Environmental Changes (cE3c), Faculty of Sciences (FCUL), University of Lisbon, Campo Grande, 1749-016 Lisbon, Portugal; 2 Instituto Superior de Agronomia, Universidade de Lisboa, Tapada da Ajuda, 1349-017 Lisbon, Portugal; 3 Centro de Investigação de Montanha (CIMO), Instituto Politécnico de Bragança, Campus de Santa Apolónia, 5300-253 Bragança, Portugal; 4 Laboratório para a Sustentabilidade e Tecnologia em Regiões de Montanha, Instituto Politécnico de Bragança, Campus de Santa Apolónia, 5300-253 Bragança, Portugal

Humans have used a multitude of wild species of plants, fungi, and animals for food and medicinal purposes. However, with the widespread establishment of industrial agriculture and globalization, the numbers of consumed plant, animal, and fungi species have significantly reduced. While in most developed or urban societies, virtually only cultivated or bred species are consumed, rural communities in many countries remain familiar with and consume many wild plant and mushroom species [1].

Wild edible plants and fungi remain an integral part of the diets of millions of people, and even in developed countries, interest in wild food has increased in recent decades; this is reflected in the popular trend of foraging or in the inclusion of wild species in the dishes of renowned restaurants. On the other hand, many wild species are relatives of wild crops with genetic potential that can be used in breeding programs, while others are undergoing domestication processes.

Wild edibles include a rich variety of plant lifeforms, such as annual and perennial herbs, shrubs, trees, lianas, and ferns, as well as saprophytic and symbiotic fungi. They are available in various ecosystems and agroecosystems and continue to play crucial roles in many food systems, providing direct and indirect resources for human nutrition and health [2]. Weeds and ruderal species that colonize disturbed sites can also be significant food sources.

Societies are nowadays faced with multiple challenges linked to climate change, global population growth, the overexploitation of natural resources, and food insecurity. Therefore, knowledge of the potential sustainable use of natural resources and their valorization sis essential in several aspects.

In less developed regions, the harvesting and consumption of wild edible plants and mushrooms remain common practices, and there is a market for a large number of species with high socio-economic value [3]. Several species of trees and, to a lesser extent, lianas and shrubs with edible parts are not removed from agricultural fields and left to become important elements of agroforestry systems. Some of these species can already be considered semi-domesticated since they are cared for and preserved by local populations.

Despite their importance, wild edible plants and fungi have received little attention from scholars, remaining largely neglected in the literature. For instance, despite the large socioeconomic importance of wild edible mushrooms across Central and Southern Africa, only very recently was the first article published on this topic in Angola [4].

On the other hand, the taxonomic identification of plant species, especially edible mushrooms, is often difficult. The clear identification of taxa with scientific names and common names; the gathering of data on local uses, properties, and socio-economic importance; and sample collection and analysis are all critically necessary.

This Special Issue of *Foods* presents leading studies from different disciplines that address edible wild plants and mushrooms. This collection of contributions addresses a diverse range of topics, from the economic potential and valorization of wild edible species as food ingredients to the diversity of Fabaceae in agroforestry systems, as well as the chemical composition and functional properties of several wild edible plants.

The characterization and understanding of the plants and fungi used in food and medicine covers various domains: (i) Their detailed characterization helps to improve the accuracy of species identification and classification. This, in turn, is crucial to ensuring precision in communicating and exchanging information regarding their properties and applications. (ii) With a comprehensive understanding of the nutritional and medicinal properties of plants and fungi, it is possible to maximize their potential. This includes an understanding of their chemical compounds and impacts on the human body, as well as determining the best methods of preparation and consumption to obtain maximum health benefits. (iii) Numerous plants and fungi are used in folk medicine due to their therapeutic attributes. By characterizing these properties, novel treatments and medications derived from natural compounds could be developed to treat a wide range of diseases and medical conditions. (iv) Given the escalating environmental degradation and biodiversity loss, the characterization and comprehension of plants and fungi used in food and medicine are imperative for their conservation and preservation. For this, identifying threatened species and implementing protective measures to ensure their long-term survival is needed. (v) Improved knowledge on these plants and fungi is also crucial for sustainable development. This entails the responsible use of natural resources, promoting sustainable harvesting practices and conserving the ecosystems that support these species.

The characterization and understanding of plants and fungi used in food and medicine might significantly contribute to enhancing human health, fostering biodiversity conservation, and boosting sustainable development. These research areas are essential to fully explore and harness the natural resources available, for human well-being.

The study by Clemente-Villalba et al. on the valorization of wild edible plants as food ingredients demonstrates the high potential of such species from nutritional, economic and social perspectives, and recommends further studies to deepen our scientific understanding of their role in the socio-economic sustainability of agriculture worldwide.

In an assessment of the Fabaceae species in cashew agroforestry systems in East Timor, Guterres et al. found 50 species commonly distributed in this area. Many of them are simultaneously used as food, fodder, and traditional medicines, while several species are predominantly used as food.

The composition of wild lingonberries, a traditional food, and an important contributor to the economic activity of non-wood forest products in the Nordic countries, were explored by Amundsen et al. During the ripening process, they observed compositional changes that might have interesting implications, namely for the berries’ harvest time.

The quality attributes and metabolic profiles of uvaia (*Eugenia pyriformis*), a native Brazilian Atlantic Forest fruit, are presented in the research of Spricigo et al. These authors found a high variability and diversity of sugars and organic acids among the analyzed accessions and forecast several possibilities of application for these fruits in different economic areas.

The variation of the chemical composition in seed-propagated populations of *Camellia ptilophylla*, a wild crop relative of tea plants, is addressed by Zheng et al. These authors consider that the species has good potential to be used as low-caffeine tea, as long as its hybridization with common tea plants is prevented.

The contributions published in this Special Issue of *Foods* represent just a small introduction to the potential of wild edible plants and mushrooms, both as ingredients in the diet of rural or urban populations, and as sources of income for families who collect and commercialize them.
